# CD86 Is an Activation Receptor for NK Cell Cytotoxicity against Tumor Cells

**DOI:** 10.1371/journal.pone.0083913

**Published:** 2013-12-11

**Authors:** Yanmeng Peng, Gaoxing Luo, Junyi Zhou, Xiaojuan Wang, Jie Hu, Yanyan Cui, Xian C. Li, Jianglin Tan, Sisi Yang, Rixing Zhan, Junjie Yang, Weifeng He, Jun Wu

**Affiliations:** 1 State Key Laboratory of Trauma, Burn and Combined Injury, Institute of Burn Research, Southwest Hospital, Third Military Medical University, Chongqing, China; 2 Chongqing Key Laboratory for Disease, Proteomics, Chongqing, China; 3 Center for Immunobiology Research, Transplant Immunology Program, Houston Methodist Hospital, Houston, Texas, United States of America; Centre de Recherche Public de la Santé (CRP-Santé), Luxembourg

## Abstract

CTLA4Ig has been successfully used in the clinic for suppression of T cell activation. However, patients treated with CTLA4Ig experienced reduced incidence of tumors than predicted, but the underlying mechanism remains unknown. In this paper, we showed that brief administration of CTLA4Ig significantly reduced tumor metastasis and prolonged the survival of host mice bearing B16 melanoma. Depletion of NK cells prior to CTLA4Ig administration eliminated the CTLA4Ig-mediated anti-tumor activity. CTLA4Ig enhanced NK cell cytotoxicity to tumor cells via up-regulation of NK cell effecter molecules CD107a and perforin *in vivo*. In addition, we demonstrated that, upon activation, NK cells could significantly increase the expression of CD86 both *in vitro* and *in vivo*, and ligation of CD86 with CTLA4Ig significantly increased the ability of NK cells to kill tumor cells. Furthermore, a human NK cell line that expressed high level of CD86 was directly activated by CTLA4Ig so that killing of tumor targets was enhanced; this enhanced killing could be inhibited by blocking CD86. Our findings uncover a novel function of CTLA4Ig in tumor immunity and suggest that CD86 on NK cells is an activating receptor and closely involved in the CTLA4Ig-mediated anti-tumor response.

## Introduction

Cytotoxic T-lymphocyte-associated antigen 4 (CTLA4) is expressed on the surface of T cells hours or days after activation and functions as a negative regulator of T cell activation. CTLA4Ig is constructed by genetically fusing the external domain of human CTLA4 to the heavy-chain constant region of human IgG1. CTLA4Ig has been shown to induce T cell tolerance by competitively binding to both CD80 and CD86 on antigen-presenting cells (APC), which prevents the binding of CD28 to its ligands CD80 and CD86 to deliver costimulatory signals to T cells[1]. Many *in vitro* and *in vivo* studies demonstrated that CTLA4Ig could be used for controlling of autoimmune diseases[2,3] and allograft rejection[4]. Both commercial products of CTLA4Ig, Abatacept and Belatecept (Bristol-Myers Squibb), have been approved by the FDA as a therapy for treating autoimmune diseases such as arthritis[5], and for controlling graft rejection[4]. One interesting aspect of CTLA4Ig is that patients treated with this fusion protein experienced much lower incidence of tumors and infectious episodes than controls[6-8]. We are interested in why immunocompetence against tumors remains while T cell activation is suppressed by CTLA4Ig. It is believed that surveillance for tumorigenesis is mediated by both adaptive and innate immune cells. Whether the innate immunity is intact when the adaptive immunity is suppressed by CTLA4Ig has not been examined. Grohmann et.al. found that CTLA4Ig could influence APC function via the interaction with B7 molecules on APC[9]. Moreover, recent studies demonstrated that resting NK cells could express CD86 and that activated NK cells express both CD80 and CD86 receptors[10,11] suggesting that NK cell function might be modulated by CTLA4Ig. These results prompted us to study the possibility as to whether CTLA4Ig can regulate tumor immune surveillance by modulating NK cell function. In the present study, we demonstrated that CTLA4Ig promotes anti-tumor immunity via enhancement of NK cell cytotoxicity to tumor cells and that ligand of CD86, but not CD80, on NK cells by CTLA4Ig is critically involved. 

## Materials and Methods

### Ethics statement

All animals were maintained under specific pathogen-free conditions and used at 6 to 8 weeks of age. The mouse protocols were approved by the Labratory Animal Welfare and Ethics Committee of the Third Military Medical University (protocol#SYXK-PLA-2007035). To ameliorate any suffering of mice observed throughout these experimental studies, mice were euthanized by CO_2_ inhalation then by following with cervical dislocation.

### Animals

C57BL/6J (B6) mice and SCID mice were purchased from the Experimental Animal Department of the Third Military Medical University, Chongqing, China. All animals were maintained under specific pathogen-free conditions and used at 6 to 8 weeks of age. 

### Reagents

CTLA4Ig was bought from R&D Systems (Minneapolis, MN, USA), and an isotype control IgG (Daclizumab) was purchased from Roche. FITC-conjugated anti-mouse NK1.1, PerCP-conjugated anti-mouse CD80, APC-conjugated anti-mouse CD86, FITC-conjugated anti-human CD56, PerCP-conjugated anti-human CD80, APC-conjugated anti-human CD86, PE-conjugated anti-human NKG2D and PE-conjugated anti-human CD336 (NKp44) were purchased from Tianjin Sungene (Tianjin, China). PE-conjugated anti-mouse perforin, APC-conjugated anti-mouse CD107A, PE-conjugated anti-mouse TNF-α and APC-conjugated anti-mouse IFN-γ were purchased from eBioscience (San Diego, CA). Purified Anti-mouse NK1.1 mAb (clone PK136) was purchased from BioXcell (USA).

### Tumor model

A melanoma tumor model was used in the study. Briefly, on day 0, 2x10^5^ B16F0 melanoma tumor cells were injected into the tail veins of either C57BL/6 mice or SCID mice. On days 0, 3 and 6, either CTLA4Ig (200 μg/mouse) or isotype control IgG (200 μg/mouse) was intravenously injected into the mice. The survival of the mice (n = 10 per group) was daily monitored over 4 weeks.

For examination of tumor lung metastasis, we euthanized tumor burden mice at day 10 after tumor injection, and the metastatic nodules on the surface of lungs were counted[12].If mice exhibited any clinical signs of distress, such as lethargic, hunched, or poor appetite, moist pellets and gel was supplied on the cage bottom, administration of subcutaneous fluids was given, or other specific treatment was given by the veterinary staff. All tumor implanted mice were euthanized on the day 26.

### NK cell depletion

B6 mice were injected intravenously with 300 μg PK136 on days -5, day -1 and day 0 refers to the time of inoculation with tumor cells. Depletion was monitored by peripheral blood draws to measure the percentage of NK cells.

### HE staining

Thin slices of lung tissue from mice bearing B16 melanoma tumor were fixed in 4% formaldehyde solution (pH 7.0) for periods not exceeding 24 h. The tissues were processed routinely for paraffin embedding, and 4 µm-thick sections were cut and placed on glass slides. The tissue samples were stained with hematoxylin and eosin.

### Preparation of cell suspensions from lungs with melanoma metastasis

B6 mice (n = 10), aged 6 weeks, were injected with 2×10^5^ B16F0 melanoma cells. Ten days after inoculation, mice were euthanized and the metastatic lung tissues were resected and treated with collagenase/hyaluronidase digestion solution buffer (0.27% collagenase, 0.025% hyaluronidase, 1% DNase, 0.01% Hepes, and 0.01% sodium pyruvate in RPMI) for 0.5 hours at 37°C. The digested lung tissue was filtered through a 40 μM cell strainer (Becton Dickinson), and a single-cell suspension was obtained that contained resident and infiltrating cells.

### NK cell isolation and culture

The mouse NK cells were isolated from B6 mice spleen or metastatic lung tissue by using an NK cell isolation kit according to the manufacturer’s instructions (Miltenyi, Germany). The purity of the isolated mouse NK cells ranged from 84% to 90%, as determined by flow cytometry.

The non-transformed immortalized human NK cell line, NK-92MI, was purchased from the American Type Tissue Collection (Manassas, VA, USA).

Both the freshly isolated mouse NK cells and the NK-92MI cell line were cultured in RPMI 1640 supplemented with 10% FBS in the presence of either 5 µg/ml CTLA4Ig or 5 µg/ml control IgG for 3 hours. 

### NK cell cytotoxicity assays

The NK sensitive YAC-1 or K562 cells were resuspended at 10^6^/ml and labeled with CFSE at a final concentration of 5 µM at 37°C. . After incubation for 10 min, YAC-1 or K562 cells were washed three times and resuspended in complete culture medium at a concentration of 2×10^4^/ml and used as target cells. Either freshly isolated mouse splenic NK cells (10^5^/well), mouse lung-infiltrating NK cells or human NK-92MI cells (10^5^/well) as the effector cells were cultured with target cells at a ratio of 5:1. After being incubated for 4 hours, all of the cells were washed twice and then stained with propidium iodide solution (50 µg/ml in PBS; Sigma) for 15 minutes. NK cell cytotoxicity was quantified immediately by flow cytometric analysis.

### Competitive binding assays

Purified anti-mouse CD80 mAb (10 μg/ml) or anti-mouse CD86 mAb (10 μg/ml) (eBioscience, USA) was added into the NK-92MI (10^6^/well) culture system either with CTLA4Ig (2.5μg/ml) simultaneously, or 2 hour earlier than CTLA4Ig (2.5μg/ml) administration. After culture with CFSE-labeled K562 target cells (2×10^5^/well) for 4 hours, NK cell cytotoxicity to K562 was determined as described above.

### Flow cytometry

For NK1.1, CD80, CD86, CD107a, NKG2D and NKp44 staining, cells were labeled with the antibodies for 30 minutes on ice, washed, fixed with PFA 4%, and resuspended in FACS EDTA. For intracellular IFN-γ, perforin and TNF-α staining, cells were stimulated with 50 ng/ml phorbol myristate acetate (PMA, SIGMA) and 500 ng/ml ionomycin (SIGMA) in the presence of 10 μg/ml Golgistop (BD), for 4 hours at 37 °C, 5% CO_2_. Cells were surface stained for 30 minutes on ice with specific antibodies against NK1.1, washed with FACS EDTA and then washed with PBS. After fixing with 4% PFA, cells were permeabilized with 0.5% saponin (SIGMA) in PBS-0.1% BSA and intracellularly stained with specific antibodies against either IFN-γ or perforin or TNF-α for 30 minutes at room temperature and then washed. Cells prepared for flow cytometry were analyzed after gating for NK^+^ viable cells using Attune™ Acoustic Focusing Cytometer (Applied Biosystems, Foster City, CA, USA). Experiments were performed a minimum of three times in an independent manner.

### Statistics

Statistical significance was evaluated using either a 2-tailed unpaired Student’s t test or nonparametric analysis if the SDs were significantly different between the 2 groups being tested, using Instat version 2.03 for Macintosh (GraphPad). The survival time of mice with tumors was compared and analyzed using the log rank test, performed by GraphPad Prism version 3.0a for Macintosh (GraphPad). Throughout the text, figures, and legends, the following terminology is used to denote statistical significance: *, *p* < 0.05; **, *p* < 0.01.

## Results

### CTLA4Ig reduced melanoma metastasis *in vivo* and prolonged host survival

**Figure 1:**
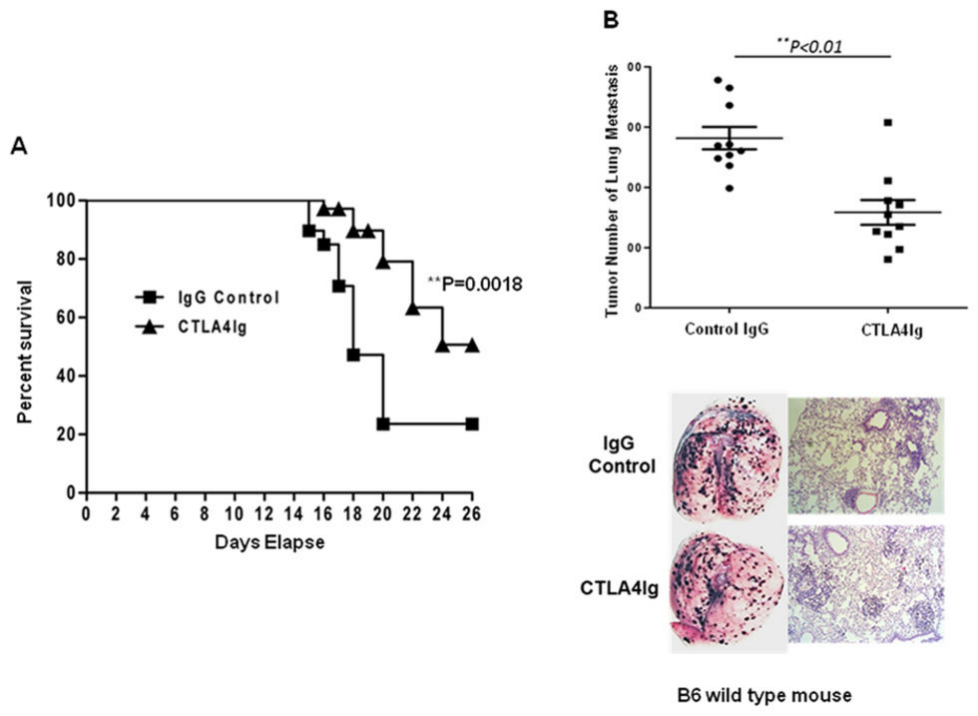
CTLA4Ig increases the survival of B6 mice bearing B16 melanoma tumors through the reduction of melanoma metastasis in B6 mice. (A) Sex- and age- matched B6 mice were injected with 2×10^5^ B16 melanoma cells via tail vein on day 0, followed by intravenous injection of either 200 μg CTLA4Ig or 200 μg isotype control IgG on days 0, 3 and 6, respectively, and the survival time of each animal was recorded. The mortality of one representative from three experiments is shown. (B) Sex- and age- matched B6 mice were injected with 2×10^5^ B16 melanoma cells via tail vein on day 0, followed by either 2 μg CTLA4Ig or 2 μg IgG control infusion via vein on days 0, 3 and day 6, respectively, and melanoma lung metastasis was assessed on day 10. The number of metastatic nodules on the lung surface, a photomicrograph and a representative H&E staining section are shown. Data are recorded as the mean ± SD (n=10), and Student’s t test is used to compare experimental and control groups. Data represents one of three independent experiments. * *p* < 0.05, ^**^ *p* < 0.01.

To define the role of CTLA4Ig in tumor immunity, sex- and age-matched B6 mice were injected with B16F0 cells (2 x 10^5^/mouse, n = 20) on day 0, followed by intravenous injection of either 200 μg CTLA4Ig (n=10) or 200 μg isotype control IgG (n=10) on days 0, 3 and 6, respectively. The survival time and the melanoma lung metastasis of each animal were monitored. The results showed that mice treated with CTLA4Ig had significantly longer survival time (Figure 1A, 20 days vs 16 days, *p*=0.0018) and significantly lower numbers of lung metastatic tumor nodules (Figure 1B, 281.6 ±18.51 vs 158.4±20.62, *p*=0.0003) than those treated with control IgG. These results suggested that CTLA4Ig induced anti-tumor activities.

### CTLA4Ig enhanced NK cell cytotoxicity *in vivo*

**Figure 2:**
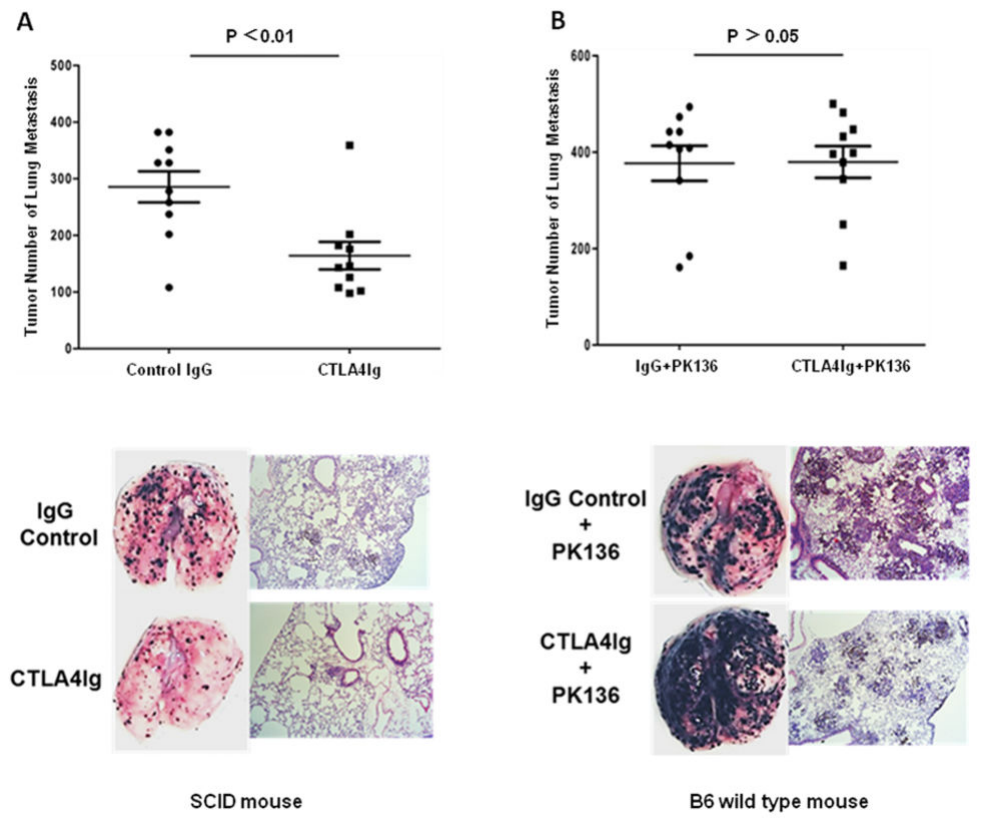
CTLA4Ig-mediated anti-tumor activity is NK cell-dependent. (A) Sex- and age- matched SCID mice were injected with 2×10^5^ B16 melanoma cells via tail vein on Day 0, followed by intravenous injection of either 200 μg CTLA4Ig or 200 μg isotype control IgG on days 0, 3 and 6, respectively, and melanoma lung metastasis was assessed on day 10. (B) Sex- and age-matched B6 mice were intravenously injected with either NK-specific antibody PK136 (n = 10) to deplete NK cells or PBS as control (n=10) on days -5, -1 and 3. The mice were then inoculated with 2×10^5^ B16 melanoma cells via tail vein on day 0, followed by intravenous injection of 200 μg CTLA4Ig on Days 0, 3 and 6. B16 melanoma lung metastasis was monitored on day 10. The number of metastatic nodules on the lung surface, a photomicrograph and a representative H&E staining section are shown. Data are recorded as the mean ± SD, and Student’s t test is used to compare experimental and control groups. Data represents one of three independent experiments. * *p* < 0.05, ^**^ *p*< 0.01.

CTLA4Ig inhibits T cell activation by blocking B7/CD28 interactions. We hypothesized that CTLA4Ig may modulate innate immune cells to boost anti-tumor immunity in our model. To test our hypothesis, sex- and age-matched SCID mice were used, and the results showed that CTLA4Ig could also significantly inhibit the lung metastasis of melanoma tumors in SCID mice (Figure 2A, 285.5±27.52 vs 164.2±24.38*, p*= 0.004). 

Because NK cells play an important role in tumor surveillance in the body, we further examined whether NK cells were involved in the CTLA4Ig-mediated anti-tumor activity. We used the PK136 depleting antibody to deplete NK cells in mice. The results showed that depletion of NK cells resulted in the abolishment of the CTLA4Ig anti-tumor protection (Figure 2B, 376.8±36.45 vs 379.5±32.84, *p*>0.05) and indicated that NK cells might play some role in the CTLA4Ig-mediated anti-tumor activity.

**Figure 3:**
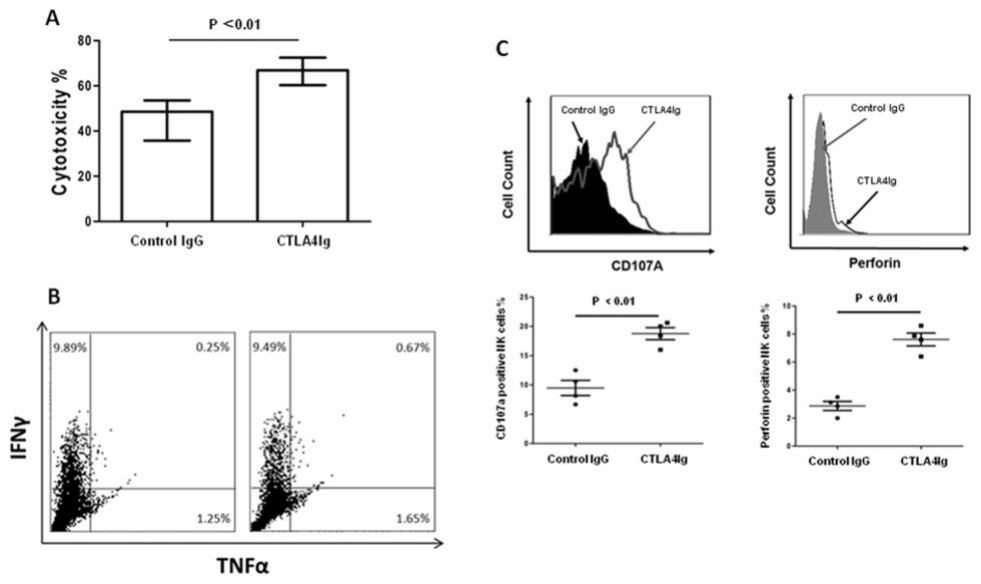
CTLA4Ig retards melanoma lung metastasis via the enhancement of NK cell cytolytic activity to tumor cells but not production of cytokine *in* *vivo*. Sex- and age- matched B6 mice were injected with 2×10^5^ B16 melanoma cells via tail vein on day 0, followed by either 200 μg CTLA4Ig or 200 μg IgG control infusion via vein on days 0, 3 and 6, respectively. The infiltrating NK cells from lung tissue on day 10 days were purified by MACS. (A) The infiltrating NK cells were co-cultured with CFSE-labeled YAC-1 cells at the ratio of 5:1 in the presence of CTLA4Ig for 6 hours. The cytotoxicity against YAC-1 was determined by flow cytometric analysis. Data are shown as the mean percentages of cytotoxicity ± SD (n=4). (B) The infiltrating NK cells were cultured with PMA, ionomycin and Golgistop for 4 hours. The expression of IFNγ and TNFα in the infiltrating NK cells was determined by flow cytometry. One representative experiment of three is shown. (C) The expression of CD107a in the infiltrating NK cells was determined directly after purified by MACS and perforin was determined after cultured with PMA, ionomycin and Golgistop for 4 hours by flow cytometry. One representative experiment of three is shown. Data represent the mean ± SD. * *p* < 0.05, ^**^ *p*< 0.01 was considered as statistically significant when compared with the control group.

Production of IFN-γ and cytotoxicity are key functions of NK cells in immune surveillance mechanisms. To assess the roles of IFN-γ and cytotoxicity in NK-dependent-CTLA4Ig-anti-tumor activity *in vivo*, the B16 melanoma mice treated with either CTLA4Ig or control IgG were sacrificed 10 days after tumor inoculation; magnetic-activated cell sorting (MACS, Miltenyi Biotec) was used to isolate the infiltrating NK cells from lung tissue for the analysis of cytolytic activity and cytokine production. The results showed that tumor-infiltrating NK cells from mice treated with CTLA4Ig possessed significantly higher cytolytic activity than those treated with control IgG (Figure 3A, from 44.2±4.2% vs 60.7±4.3%, *p*=0.007), but there were no significant differences in IFNγ and TNFα production in NK cells between the CTLA4Ig group and control IgG group (Figure 3B). These results suggest that CTLA4Ig retards tumor metastasis by enhancing the NK cell cytotoxicity to tumor cells *in vivo*.

Because the degranulation marker CD107a and effector molecule perforin are also closely associated with the NK cell cytotoxicity to tumor cells, we examined the expression of the molecules in tumor infiltrating NK cells of mice treated with either CTLA4Ig or control IgG. The results showed that there were significantly higher numbers of perforin-producing and CD107a-positive NK cells in CTLA4Ig-treated mice than in control IgG-treated mice (Figure 3C, perforin: 8.47±0.83% vs 3.43±0.37%, *p*=0.0054; CD107a: 18.75±1.03% vs 9.48±1.29%, *p*=0.0014). These results clearly showed that the cytotoxicity of the infiltrating NK cells was markedly enhanced in the process of CTLA4Ig-mediated anti-tumor.

### CTLA4Ig stimulated NK cell cytolytic activity to tumor cells

**Figure 4:**
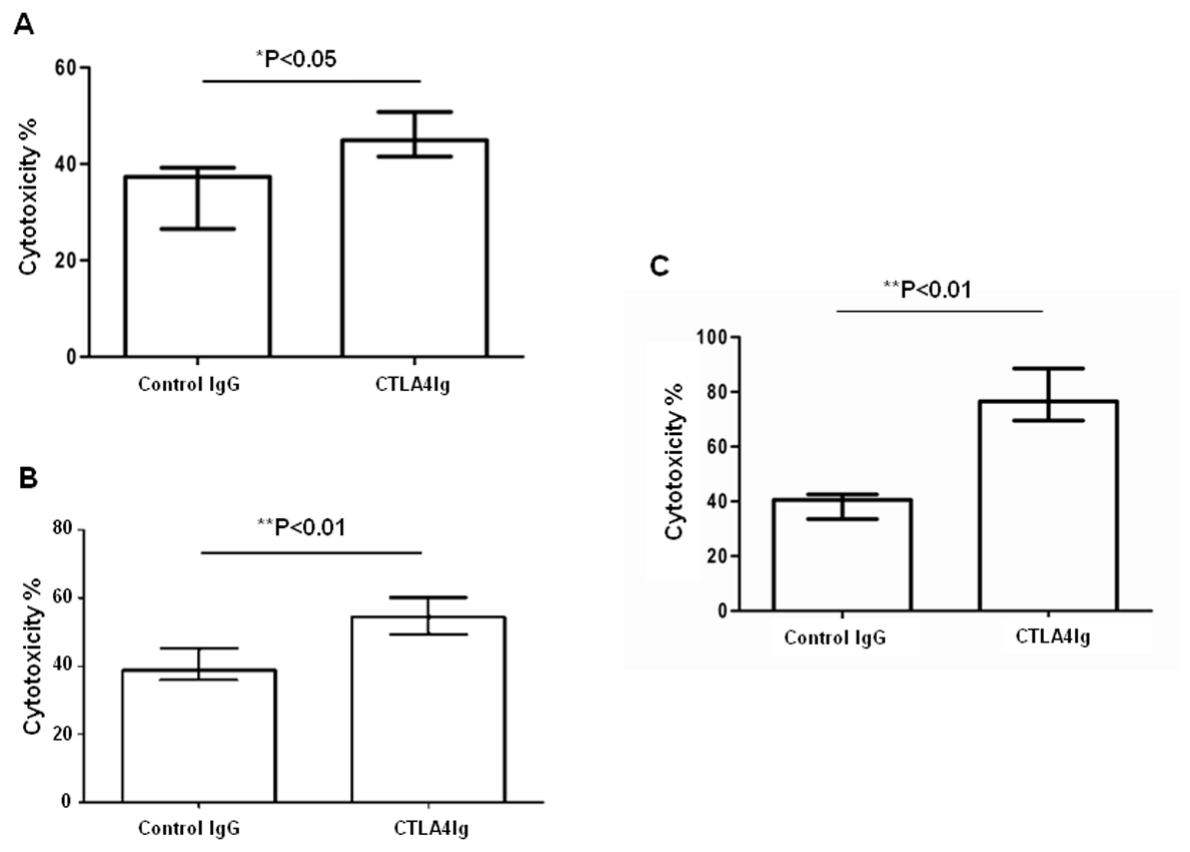
CTLA4Ig directly stimulates NK cells to enhance NK cell cytolytic activity against tumor cells. (A) Freshly isolated B6 mouse splenic NK cells were co-cultured with YAC-1 cells at the ratio of 5:1 in the presence of 5 µg/ml of CTLA4Ig for 6 hours *in* *vitro*. The cytotoxicity against YAC-1 was determined by flow cytometry. Data are shown as the mean percentages of cytotoxicity ± SD (n=4). (B) Sex- and age- matched B6 mice were injected with 2×10^5^ B16 melanoma cells via tail vein on day 0. On day 3, the infiltrating NK cells from lung tissue of mice bearing B16 melanoma tumor were purified by MACS and co-cultured with CFSE-labeled YAC-1 at the ratio of 5:1 in the presence of either 5 µg/ml CTLA4Ig or 5 μg/ml control IgG for 6 hours. The cytotoxicity against YAC-1 was determined by flow cytometry. Data are shown as the mean percentages of cytotoxicity ± SD (n=3). (C) NK-92MI cells were cultured with CFSE-labeled K562 at the ratio of 5:1 in the presence of 5 μg/ml CTLA4Ig for 6 hours. The cytotoxicity against K562 was determined by flow cytometry. Data are shown as the mean percentages of cytotoxicity ± SD (n=3). * *p* < 0.05, ^**^ *p* < 0.01 was considered as statistically significant when compared with the control group.

Although administration of CTLA4Ig enhances the tumor killing capacity of NK cell *in vivo*, it is still unclear if CTLA4Ig can act directly on NK cells. To assess the direct effect of CTLA4Ig on NK cells, we first examined the role of CTLA4Ig in NK cell cytotoxicity to tumor cells *in vitro*. Mouse splenic NK cells were purified by MACS and co-cultured with either CTLA4Ig or control IgG to analyze the cytolytic activity to YAC-1 cells. Compared to control IgG, CTLA4Ig could significantly enhance NK cell cytotoxicity to YAC-1 cell *in vitro* (Figure 4A, from 40.1±2.3% to 48.3±3.3%, *p*=0.0313). Then, we checked the effect of CTLA4Ig on NK cell cytotoxicity to tumor cells *ex vivo*. The tumor-infiltrating NK cells were purified from lungs of mice bearing B6 melanoma tumor and co-cultured with either CTLA4Ig or control IgG to analyze the cytolytic activity to YAC-1 cells. Compared to control IgG, CTLA4Ig significantly enhanced the cytotoxicity of the infiltrating NK cells *ex vivo* (Figure 4B, 40.2±3.2% vs 58.7±4.3%, *p*=0.0007).

Because the NK cells isolated using MACS were not highly purified (the purity was less than 90%), it was possible that the CTLA4Ig was acting on other cell types in the culture. To address this issue, we used a human NK cell line, NK-92MI, as the effector cells to check the possible direct role of CTLA4Ig in regulating NK function. The results showed that cytotoxicity of NK-92MI cells to K562 tumor cell was significantly greater increased in the presence of CTLA4Ig than that in the presence of control IgG *in vitro* (Figure 4C, 57.5±4.1% vs 38.9±2.7%, *p*<0.0001). These data suggest that CTLA4Ig directly stimulates NK cell cytotoxicity.

### CD86 is the target molecule in CTLA4Ig-mediated enhancement of NK cytolytic activity

**Figure 5:**
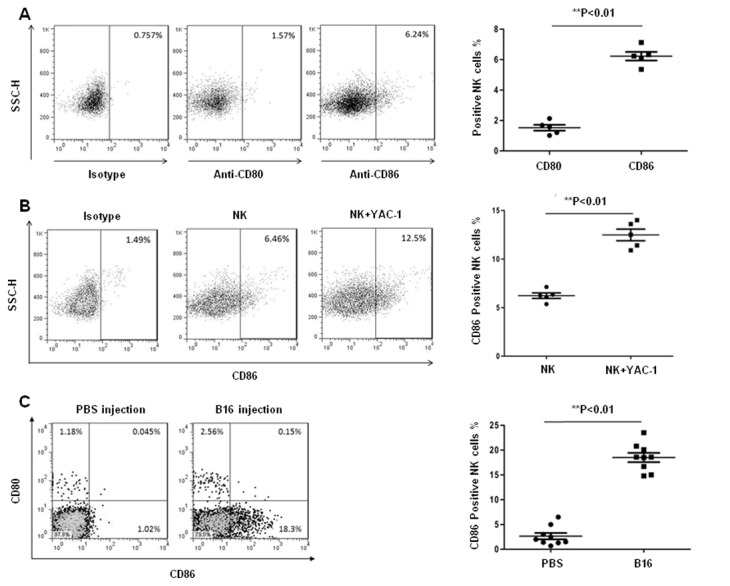
The expression of CD80 and CD86 on either physiological or tumor-activated NK cells. (A) The expression of CD80 and CD86 on freshly isolated mouse splenic NK cells was analyzed by flow cytometry. (B) The freshly isolated mouse splenic NK cells were cultured *in* *vitro* with or without YAC-1 cells for 24 hours. The expression of CD86 was assessed by flow cytometry. (C) Sex- and age-matched B6 mice were inoculated with 2×10^5^ B16 melanoma cells via tail vein on day 0. Three days after inoculation of B16 melanoma cells or PBS, the lung-infiltrating NK cells were purified by MACS, and the expression of CD80 and CD86 analyzed by flow cytometry. All experiments were repeated three or four times. * *p* < 0.05, ^**^ *p* < 0.01 was considered as statistically significant when compared with the control group.

Based on the fact that CTLA4Ig binds with high affinity to CD80/CD86, we hypothesized that CD80 or CD86 might play in role in the ability of CTLA4Ig to enhance NK cell functions against tumor metastasis. To understand the expression of CD80 and CD86 on physiological NK cells of B6 mouse, the CD80/CD86 expression on NK cells was examined by flow cytometry. The results showed that prior to activation, mouse NK cells had 6% expression of CD86 and little CD80 expression (Figure 5A). The expression of CD86 on NK cells was significantly increased following activation with both YAC-1 tumor cells (Figure 5B, from 6.2% to 12.6%) and cytokine IL-15 (Figure S2) *in vitro*. Furthermore, we detected the expressions of CD86 and CD80 on the tumor-infiltrating NK cells *in vivo* 10 days after injection of B16 melanoma cells. The data showed that injection of B16 melanoma cells could significantly increase the expression of CD86, but not CD80, on infiltrating NK cells (Figure 5C, from 2.5% to 18.6%). These results indicated that NK cells could significantly increase the expression of CD86 upon tumor cell stimulation and that CTLA4Ig possibly activates NK cells via ligation of CD86 on the activated NK cells.

**Figure 6:**
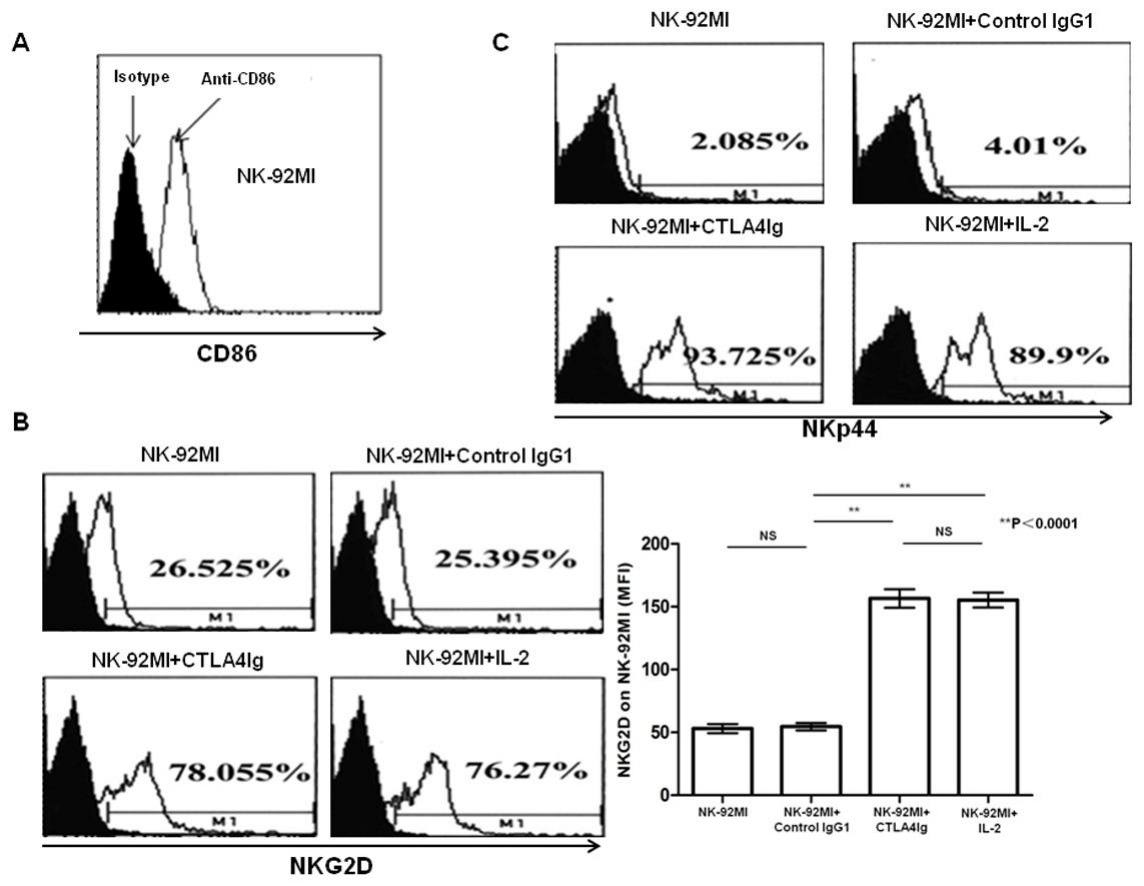
CTLA4Ig directly activates NK-92MI cells to induce marked expression of the NK cell effector molecules NKG2D and NKp44. (A) Expression of CD86 on NK-92MI cells was analyzed by flow cytometry. One representative experiment of four is shown. (B) Flow cytometry was used to assess the expression of the activation receptors NKG2D on NK-92MI cells treated with either CTLA4Ig or isotype IgG or IL-2 for 24 hours. One representative experiment of four is shown. ^**^ *p* < 0.0001 was considered as statistically significant when compared with the control group. (C) Flow cytometry was used to assess the percentage of NKp44 positive NK-92MI cells after treated with either CTLA4Ig or isotype IgG or IL-2 for 24 hours. One representative experiment of four is shown.

**Figure 7:**
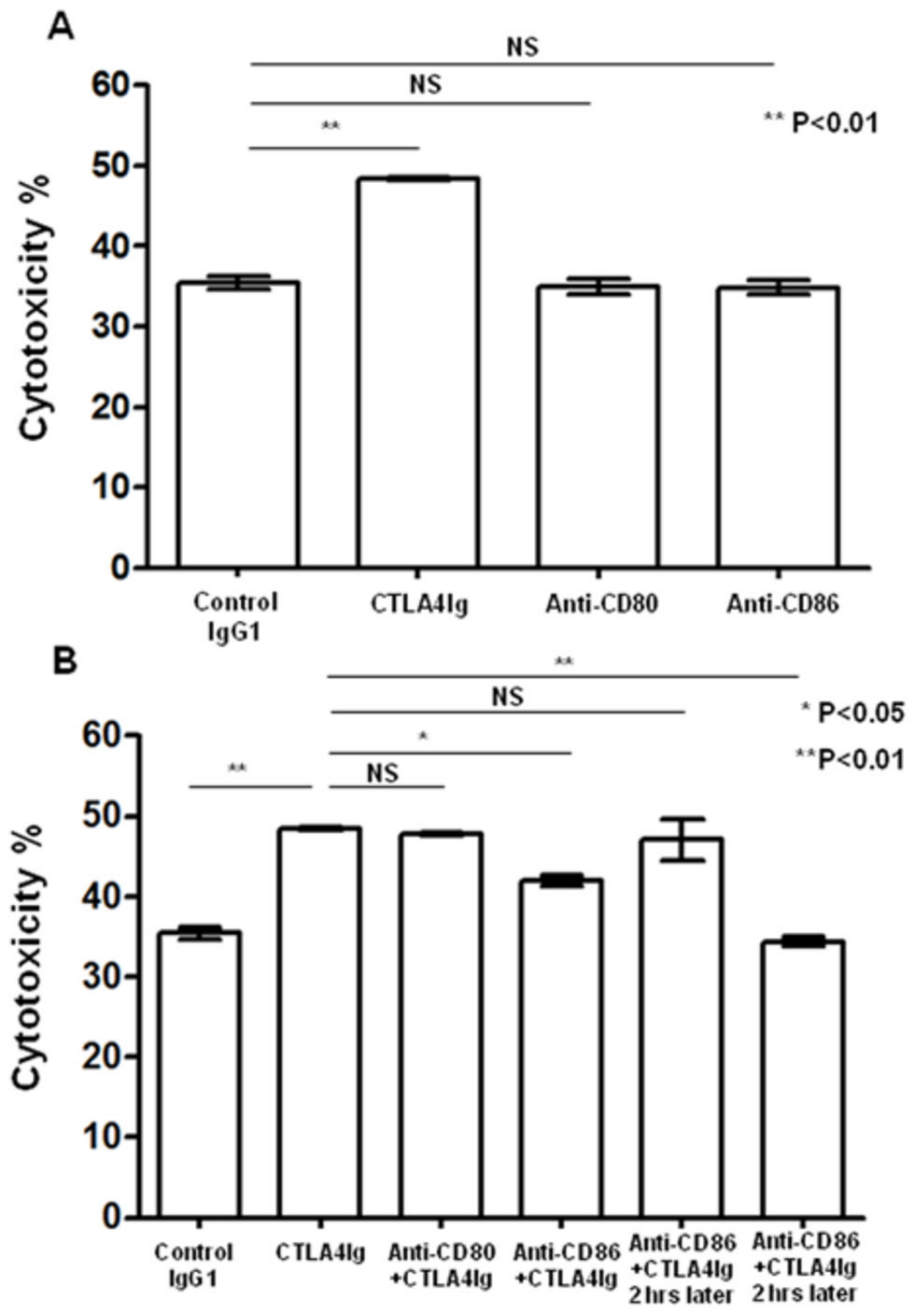
CD86 on NK cells contributes to CTLA4Ig-mediated NK cytotoxicity. (A) NK-92MI cells were cultured with CFSE-labeled K562 cells at the ratio of 5:1 in the presence of either 5 μg/ml soluble CTLA4Ig, soluble IgG1, anti-CD80 antibody, or anti-CD86 antibody for 6 hours. Cytotoxicity against K562 was determined by flow cytometry. Data are shown as the mean percentages of cytotoxicity ± SD (n=3). (B) NK-92MI cells were cultured for 6 hours with CFSE-labeled K562 cells at the ratio of 5:1 in the presence of either simultaneous addition of 5 μg/ml soluble IgG1, soluble CTLA4Ig, anti-CD80 antibody+5 μg/ml soluble CTLA4Ig, anti-CD86 antibody+5 μg/ml soluble CTLA4Ig, or treatment, two hours earlier, with anti-CD80 antibody+5 μg/ml soluble CTLA4Ig, anti-CD86 antibody+5 μg/ml soluble CTLA4Ig. The cytotoxicity against K562 was determined by flow cytometry. Data are shown as the mean percentages of cytotoxicity ± SD (n=3). All experiments were repeated 3 times. * *p* < 0.05, ^**^ *p* < 0.01 was considered as statistically significant when compared with the control group.

To test the role of CD86 in regulating NK cell function, we used NK-92MI cells that constitutively expressed CD86 (Figure 6A, more than 70.6%), but not CD80 (data not shown), as effector cells to test the effect of CD86 ligation with CTLA4Ig *in vitro*. As shown in Figure 6B, CD86 ligation with CTLA4Ig up-regulated NKG2D expression on the cell surface in terms of percentage and intensity (as measured by MFI). As compared to cells without CD86 ligation (MFI values 54.5±2.84), there was a marked increase in NKG2D MFI (MFI values 156.5±7.44) (p<0.0001). Similarly, CD86 ligation also substantially induced NKp44 expression as compared the controls as shown in Figure 6C (91.1±2.48% vs 3.26±0.286%, p<0.0001). For both NKG2D and NKp44, their levels of expression upon CD86 ligation were comparable to those seen following IL-2 stimulation (Figure 6B and 6C), which suggested that CD86 on NK cells might be involved in the process of regulating NK cell function. Based on these data, either anti-CD80 antibody or anti-CD86 antibody was used to stimulate NK-92MI to further confirm the role of CD80 or CD86 in NK cell function. As there was little expression of CD80 on the NK-92MI cell surface (data not shown), as expected, no effect of anti-CD80 antibody on NK-92MI cell cytotoxicity was found (Figure 7A, anti-CD80 vs Isotype IgG, *p*>0.05; anti-CD86 vs Isotype IgG, *p*>0.05). Interestingly, anti-CD86 antibody failed to enhance NK cell cytotoxicity (Figure 7A, anti-CD80 vs Isotype IgG, *p*>0.05; anti-CD86 vs Isotype IgG, *p*>0.05); this might have been because anti-CD86 antibody cannot crosslink CD86 on their own. Therefore, an anti-CD86 antibody was used to compete for CD86 molecules on NK-92MI with CTLA4Ig. The results showed that when anti-CD86 antibody, together with CTLA4Ig, was added into the NK-92MI cell culture system, the CTLA4Ig-mediated NK-92MI cell activation was partly blocked (Figure 7B anti-CD86+CTLA4Ig vs CTLA4Ig, *p*=0.026). When anti-CD86 antibody was added 2 hours earlier than CTLA4Ig, CTLA4Ig-mediated NK-92MI cell cytotoxicity was completely abolished (Figure 7B anti-CD86+CTLA4Ig 2 hours later vs isotype IgG1, *p*=0.004). These data from the competition experiments clearly demonstrated that CD86 rather than CD80 on NK cells was involved in the enhancement of NK cell cytotoxicity to tumors. 

## Discussion

CTLA4Ig blocks CD28 costimulatory signaling to T cells by competitively binding to CD80/CD86, and it has marked effect in damping T cell-mediated immune responses. In fact, CTLA4Ig has been used in the clinic to treat several immune-mediated diseases with favorable outcomes. Interestingly, clinical trials show that the incidence of tumors are lower than expected[13,14], which is incompatible with the generally held view that immune suppressive reagents, such as cyclosporine A[15,16], produce higher tumor incidence due to broad immunosuppression. Because the immune system consists of innate and adaptive immunity and CTLA4Ig has been well demonstrated to be the inhibitor of T cell activation, we were interested in how the innate immune system responds to CTLA4Ig treatment. To our surprise, instead of inhibition, a brief administration of CTLA4Ig could significantly reduce tumor metastasis and prolong the survival of hosts bearing B16 melanoma tumor (Figure 1A and 1B). We showed that this effect was mediated by NK cell activation. By using SCID mice (Figure 2A) and NK depletion experiments (Figure 2B), we demonstrated that NK cells were critical for CTLA4Ig-mediated anti-tumor activity.

Our data suggest that CTLA4Ig plays an anti-tumor role via the enhancement of NK cell activity. Based on the analysis of tumor-infiltrating NK cells in mice bearing melanomas, we revealed that CTLA4Ig could significantly promote the cytolytic activity of the infiltrating NK cells *in vivo* (Figure 3A) via the up-regulation of NK cell cytotoxicity (Figure 3C) but not through the production of IFN-γ and TNF-α (Figure 3B). Through the killing assays *in vitro* (Figure 4A) and *ex vivo* (Figure 4B), we proved that CTLA4Ig could significantly enhance NK cell cytotoxicity to tumor cells as well. We consistently observed that CTLA4Ig could significantly enhance the cytotoxicity of NK cells from human peripheral blood (Figure S1). Moreover, by using the human NK cell line NK-92MI, we confirmed that CTLA4Ig could directly activate NK cells to induce increased expression of NKG2D and NKp44, as measured by MFI and percentage of cells positive (Figure 6B and 6C), and CD86 ligated NK cells showed significantly enhanced cytolytic activity to tumor cells (Figure 4C) *in vitro*. These data clearly demonstrated that CTLA4Ig, a T cell inhibitor, was a strong NK cell cytotoxicity enhancer. Importantly, this divergent capacity of CTLA4Ig on innate and adaptive immunity can inhibit the adaptive immunity for controlling unwanted T cell activation, but on the other hand, stimulate innate immunity against tumorigenesis.

As ligands of CTLA4Ig, CD80 and CD86 are proteins that are highly expressed on APCs that provide the costimulatory signals necessary for T cell activation and survival[17,18]. Although some reports showed that B7 molecules were involved in regulating APCs[9,19], the precise role of B7 molecules in regulating NK cell functions is still unknown. Previous studies showed that physiological NK cells could express CD86 but not CD80[10,11]. In agreement with those studies, we showed here that approximately 6% NK cells were CD86 positive, but few NK cells (i.e., <1%) were CD80 positive (Figure 5A). One previous study reported that NK cells activated with IL-2 could highly express CD80 and CD86[10,11]. Similarly, we found that IL-15 could stimulate NK cells to significantly increase the expression of CD80 and CD86 *in vitro* (Figure S2). Using tumor cells rather than IL-15 and IL-2 as stimulators also induced significant expression of CD86, but not CD80, on NK cells, both *in vitro* and *in vivo* (Figures 5B and 5C). This suggested that CD86, but not CD80, might be involved in the CTLA4Ig-mediated anti-tumor activity by regulating NK cell function. Some reports demonstrated that CTLA4Ig could regulate the function of APCs through the binding of CD86 on the cell surface[9]. Our data, together with other reports, indicate that CTLA4Ig could serve as a stimulator to promote the function of NK cells through the ligation of CD86 on NK cells. Although previous studies demonstrated that NK cells could express CD28 and be activated following interaction with CD80/CD86 and that this activation could be blocked by CTLA4Ig and thus lead to an inhibition of NK cell function[20-22], the role of CD80/CD86 on NK cells was unclear. To address this issue, NK-92MI cells that spontaneously expressed high level of CD86 (Figure 6A, 70.6%), were used as effector cells in the presence of CTLA4Ig to assess the role of CD86 on NK cells in regulating NK function. Our data showed that ligation of CD86 with CTLA4Ig *in vitro* could significantly enhance the tumor-killing ability of NK-92MI cells to K562 tumor cells (Figure 4C), which suggested that CD86 on NK cells might be involved in the process of regulating NK cell function. Based on these data, either anti-CD80 antibody or anti-CD86 antibody was used to stimulate NK-92MI to further confirm the role of CD80 or CD86 in NK cell function. As there was little expression of CD80 on the NK-92MI cell surface (data not shown), as expected, no effect of anti-CD80 antibody on NK-92MI cell cytotoxicity was found (Figure 7A). Interestingly, anti-CD86 antibody failed to enhance NK cell cytotoxicity (Figure 7A); this might have been because that anti-CD86 antibody cannot crosslink CD86 on their own. Therefore, an anti-CD86 antibody was used to compete for CD86 molecules on NK-92MI with CTLA4Ig. The results showed that when anti-CD86 antibody, together with CTLA4Ig, was added into the NK-92MI cell culture system, the CTLA4Ig-mediated NK-92MI cell activation was partly blocked (Figure 7B). If anti-CD86 antibody was added 2 hours earlier than CTLA4Ig, which meant that the anti-CD86 antibody had pre-occupied the CD86 molecules on NK cells, CTLA4Ig-mediated NK-92MI cell cytotoxicity was completely abolished (Figure 7B). These data from the competition experiments clearly demonstrated that CD86 rather than CD80 on NK cells was involved in the enhancement of NK cell cytotoxicity to tumors. 

NK cell cytotoxicity could be activated via FcR signaling that is referred to as antibody-dependent cellular cytotoxicity (ADCC)[23]. However, the enhanced NK cell cytotoxicity by CTLA4Ig is independent of FcR cross-linking, as CTLA4Ig similarly enhanced the killing activity of NK-92MI cells that do not express FcR, Thus, the results clearly showed that CTLA4Ig enhanced the NK cell cytolytic activity in an ADCC-independent manner.

In summary, we have demonstrated that CTLA4Ig can have different effects on innate and adaptive immunity. CTLA4Ig inhibits T cell activation via competitive binding to CD80/CD86 on APCs, which deprives T cells of CD28 mediated costimulation. On the other hand, it enhances NK cell function via ligation of CD86 on NK cells. This finding may have important clinical implications.

## Supporting Information

Figure S1 **CTLA4Ig significantly enhanced human NK cell cytolytic activity to K562 tumor cells.** Freshly isolated human peripheral blood NK cells cultured with K562 cells at the ratio of 5:1 in the presence of either 5μg/ml CTLA4Ig or 5μg/ml control IgG for 6 hours. The cytotoxicity against K562 was determined by FACS.
(TIF)

Figure S2 **The expression of CD80 and CD86 on IL-15-activated NK cells.** The freshly isolated mouse splenic NK cells were cultured *in* *vitro* with or without 5ng/ml IL-15 for 24 hours. The expression of CD86 and CD80 was assessed by FACS.
(TIF)
