# Supplementary figures and images for: CD86 Is an Activation Receptor for NK Cell Cytotoxicity against Tumor Cells
Source: PLoS One. 2013 Dec 11;8(12):e83913. doi: 10.1371/journal.pone.0083913 (PMC3859666; doi:10.1371/journal.pone.0083913)

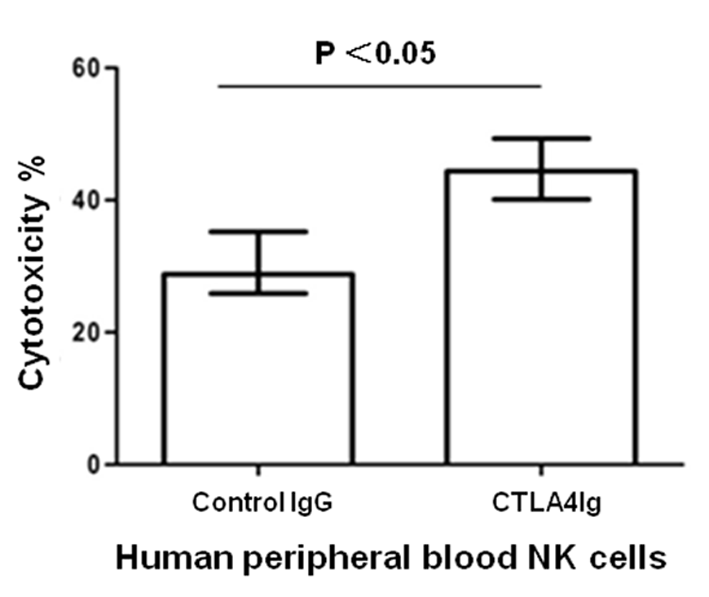

Supplement: Figure S1 — CTLA4Ig significantly enhanced human NK cell cytolytic activity to K562 tumor cells. Freshly isolated human peripheral blood NK cells cultured with K562 cells at the ratio of 5:1 in the presence of either 5μg/ml CTLA4Ig or 5μg/ml control IgG for 6 hours. The cytotoxicity against K562 was determined by FACS. (TIF) [file pone.0083913.s001.tif]

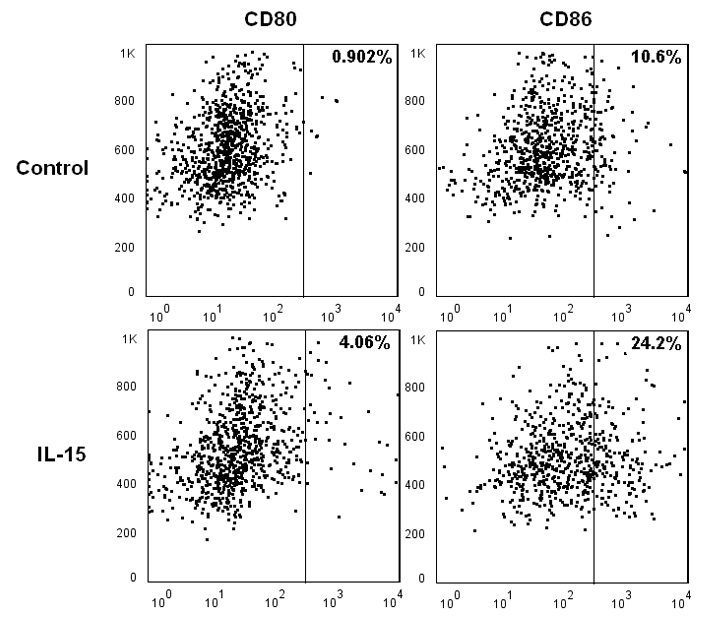

Supplement: Figure S2 — The expression of CD80 and CD86 on IL-15-activated NK cells. The freshly isolated mouse splenic NK cells were cultured in vitro with or without 5ng/ml IL-15 for 24 hours. The expression of CD86 and CD80 was assessed by FACS. (TIF) [file pone.0083913.s002.tif]
